# Molecular Mechanism for PACAP 38-Induced Neurite Outgrowth in PC12 Cells

**DOI:** 10.1155/2021/2522454

**Published:** 2021-08-07

**Authors:** Junko Shibato, Fumiko Takenoya, Takahiro Hirabayashi, Ai Kimura, Michio Yamashita, Ichiro Takasaki, Randeep Rakwal, Seiji Shioda

**Affiliations:** ^1^Global Research Center for Innovative Life Science, Peptide Drug Innovation, School of Pharmacy and Pharmaceutical Sciences, Hoshi University, 4-41 Ebara 2-chome, Shinagawa, Tokyo 142-8501, Japan; ^2^Department of Physiology and Molecular Sciences, Division of Comprehensive and Fundamental Pharmaceutical Education and Research, School of Pharmacy and Pharmaceutical Sciences, Hoshi University, 2-4-41 Ebara, Shinagawa-ku, Tokyo 142-8501, Japan; ^3^Department of Pharmacology, Graduate School of Science and Engineering, University of Toyama, Toyama, Japan; ^4^Faculty of Health and Sport Sciences, University of Tsukuba, 1-1-1 Tennodai, Tsukuba, Ibaraki 305-8574, Japan

## Abstract

The present research investigates the molecular mechanism of neurite outgrowth (protrusion elongation) under pituitary adenylate cyclase-activating polypeptide (PACAP) 38 treatments using a rat adrenal-derived pheochromocytoma cell line—PC12. This study specifically looks into the regulation of PACAP38-induced collapsing response mediator protein 2 (CRMP2) previously identified in a mouse brain ischemia model and which could be recovered by PACAP38 treatment. Previously, DNA microarray analysis revealed that PACAP 38-mediated neuroprotection involved not only CRMP2 but also pathways related to glycogen synthase kinase-3*β* (GSK-3*β*) and other signaling components. Thus, to clarify whether CRMP2 acts directly on PACAP38 or through GSK-3*β* as part of the mechanism of PACAP38-induced neurite outgrowth, we observed neurite outgrowth in the presence of GSK-3*β* inhibitors and activators. PC12 cells were treated with PACAP38 being added to the cell culture medium at concentrations of 10^−7^ M, 10^−8^ M, and 10^−9^ M. Post PACAP38 treatment, immunostaining was used to confirm protrusion elongation of the PC12 cells, while RT-PCR, two-dimensional gel electrophoresis in conjunction with Western blotting, and inhibition experiments were performed to confirm the expression of the PACAP gene, its receptors, and downstream signaling components. Our data show that neurite protrusion elongation by PACAP38 (10^−7^ M) in PC12 cells is mediated through the PAC1-R receptor as demonstrated by its suppression by a specific inhibitor PA-8. Inhibitor experiments suggested that PACAP38-triggered neurite protrusion follows a GSK-3*β*-regulated pathway, where the AKT and cAMP/ERK pathways are involved and where the inhibition of Rho/Roc could enhance neurite protrusion under PACAP38 stimulation. Although we could not yet confirm the exact role and position of CRMP2 in PACAP38-mediated PC12 cell elongation, it appears that its phosphorylation and dephosphorylation have a correlation with the neurite protrusion elongation through the interplay of CDK5, which needs to be investigated further.

## 1. Introduction

The physiologically active neuropeptide pituitary adenylate cyclase-activating polypeptide (PACAP) belonging to the vasoactive intestinal polypeptide (VIP)/glucagon/secretin family as a 27 or 38 amino acid residue (PACAP27 or PACAP38) exhibits a diverse array of biological functions [1–3]. The bioactivity of PACAP is multifunctional, and it is thought to function as a neurotransmitter/regulator in addition to its hormonal action. It is involved in the differentiation and survival maintenance of nerve cells and activation of the neurosecretory system in the central and peripheral nervous systems and regulates nerve synaptic plasticity, differentiation of neural precursor cells, and glucose-dependent insulin secretion. PACAP has been reported to have a promoting effect, a cell death inhibitory effect during cerebral ischemia, and a cytoprotective effect [2, 4–17]. Generally, the spinal cord and brain, as critical structures of the central nervous system, do not recover once they are damaged, and this is the case for the damaged nerve itself in a variety of diseases resulting from causes such as cerebral infarction, traffic accidents, and spinal cord injury caused by falls. No cure has yet been established to restore this nerve damage and the conditions (diseases/disorders) arising from it.

PACAP is recognized by receptors in the cell membrane, and like the VIP family members, it shares two common G protein-coupled receptors (GPCRs) VPAC1 R and VPAC2 R. However, PACAP has particular affinity for PAC1-R that is said to be more than 1000 times higher than those of the other two [4, 18, 19]. Studying PACAP and clarifying its function by examining a myriad of downstream (of its receptor) primary and secondary signaling pathways and network linkages may lead to confirmation of the role of PACAP in numerous cellular processes as well as contribute to the development of therapeutic drugs for diseases and disorders [5, 6]. Neuroprotection has attracted a lot of interest in brain research, and our research group has been investigating PACAP effects in the ischemic brain using high-throughput “omics” techniques. One of the high-throughput technologies is the whole genome expression profiling approach. This approach provides a snapshot of almost all (depending on sample quality and experimental design) the molecular events occurring at the level of the gene (i.e., transcriptome) at a particular instance and biological location. Using this genomic approach, we have since established a rigorous and standardized DNA microarray-based protocol to identify with high confidence the transcriptome in a mouse model of permanent middle cerebral artery occlusion (PMCAO) [16]. Additionally, we also utilized a gel-based proteomics approach using the same PMCAO mouse model to identify a collapsing response mediator protein 2 (CRMP2) in the ischemic brain tissue sample following intraventricular administration of PACAP38 [17, 20].

CRMP2, initially called CRMP-62, was first identified in 1995 using the *Xenopus* oocyte expression system [21]. CRMPs are multifunctional adapter proteins/microtubule-related proteins, highly expressed in the brain/central nervous system [22, 23]. In particular, among other functions of CRMP2, it is known to be important for the determination of neuronal polarity and process elongation associated with process injury and neuronal death, neuropathy, and cerebral ischemia [24–30]. These studies led us to hypothesize the yet-to-be-demonstrated role for CRMP2 involvement in the neurite protrusion elongation action of PACAP, including the possible molecular mechanisms underlying such a function if it exists. The CRMP2 induces protrusion formation in the cultured hippocampus, and its action has been reported to be inactivated through phosphorylation by glycogen synthase kinase-3*β* (GSK-3*β*) [31, 32]. Furthermore, in addition to GSK-3*β*, CRMP2 impairs tubulin binding ability by being phosphorylated via kinases such as the cyclin-dependent kinase 5 (Cdk5) [33] and the Rho-associated protein kinase (ROCK) [34]. Previous research has also revealed an involvement of CRMP2 phosphorylation in axon guidance and growth cones [35, 36]. However, little is known about the mechanism governing dendrite guidance and patterns by PACAP and it is unclear how CRMP2 phosphorylation is involved in neurite protrusion elongation.

Therefore, in this report, we specifically aimed to unravel the molecular mechanism behind the neurite protrusion elongation action of PACAP38 and therein the involvement of CRMP2 in a PC12 cell model [37], which are cultured immortalized cells known to differentiate into neurons by NGF and PACAP stimulation. Further, we hoped to find new evidence into the mechanism by which PACAP38 promotes the suppression of the phosphorylation of CRMP2 and thereby gain insight into the pathways that might be involved in the treatment of neuron/nerve damage and for neurodegenerative diseases/disorders.

## 2. Cell Model and Methods

### 2.1. Cell Culture

PC12 cells (RCB0009) were obtained from the RIKEN Cell Bank (Japan). Cells were cultivated in RPMI medium 1640 (ATCC Modification, Thermo Fisher) in a CO_2_ incubator (37°C, 5%) with 5% fetal bovine serum (FBS) (16140063, Gibco), 10% horse serum (HS) (H1138, SIGMA), and antibiotics (penicillin-streptomycin, P4458, Sigma). Cellmatrix Type IV (Nitta Gelatin Inc.) was used to coat the culture dish. Institutional ethical approval was not required for this study. Experiments as performed below were repeated multiple times, (usually *n* = 6) as indicated in the figure legends, and the data were presented as the mean ± SD. In the inhibitor experiment, DMSO was adjusted to a final concentration of 0.0001% (*v*/*v*) and the medium was used as a control. Except for the inhibitor experiments, SDW was added at 1% of the medium and used as a control.

### 2.2. Measurement of Neurite Protrusion Elongation

PC12 cells were adjusted to 1 × 10^3^ to 5 × 10^3^ cells/well in a 96-well plate, and after about 6 hours, PACAP (PACAP38, 052-05 Phoenix Pharmaceuticals Inc.) was added to the cell culture medium at concentrations of 10^−7^ M, 10^−8^ M, and 10^−9^ M after confirming complete adhesion to the plate. The cells were observed as a phase-contrast image using an optical microscope BZ-X710 (Keyence) over time at 17, 72, and 144 hours after the addition of PACAP38. Cells with a neurite outgrowth of 20 *μ*m or more from the image were counted as 1. Cells were counted using ImageJ software (National Institutes of Health, Bethesda, MD, USA). The percentage of cells with a neurite outgrowth of 20 *μ*m or greater was obtained by dividing by the total number of cells per image. Finally, the results for all fields examined for each condition were averaged (*n* = 6) to calculate the number of neurite outgrowth cells per condition.

### 2.3. Immunostaining

PC12 cells were seeded in a cover glass chamber (5222-004, Iwaki), and after confirming adhesion, PACAP was added to a working concentration of 10^−7^ M and the cells were cultured for 3 days. After removing the medium, the cells were washed with PBS and fixed with a 4% PFA solution. After washing again, the cells were treated with PBS solution containing 0.1% Triton X 100 for 10 minutes. After treatment and washing with PBS, blocking was carried out with PBS solution containing 3% bovine serum albumin (BSA) (Sigma-Aldrich) and 0.1% Tween 20, followed by incubation using a mouse anti-*α*-tubulin antibody (Abcam Inc., 1 : 1000) overnight at 4°C. The cells were then washed with PBS and crossreacted with Alexa 594 donkey anti-mouse IgG (Invitrogen, 1: 1000) as a secondary antibody at room temperature for 60 minutes. After washing again with PBS, Alexa Fluor 488-labeled rabbit anti-Neu-N antibody (Abcam, 1 : 50) was crossreacted overnight at 4°C. Postrinsing with PBS, nuclear-specific staining was performed with DAPI (D 1306; Thermo Fisher Scientific, 1: 10000) for 3 minutes at room temperature and the cells were finally washed with PBS and sealed. After drying, the cells were observed under a fluorescence microscope BZ-X 710 (Keyence).

### 2.4. Total RNA Extraction and RT-PCR

Total RNA was extracted from PC12 cells cultured in a 10 cm culture dish using the RNeasy Mini Kit (74104, QIAGEN). After synthesizing cDNA using the AffinityScript QPCR cDNA Synthesis Kit (600559, Agilent), PCR reaction was performed using EmeraldAmp PCR Master (RR300A, TaKaRa). PCR was carried out at an initial denaturation at 95°C for 5 minutes, and the postfinal cycle extension was performed at 72°C for 10 minutes. The primers and cycling conditions used are shown in Table 1. PCR products were separated on a 1.6% agarose gel and visualized with ethidium bromide staining under UV light.

### 2.5. PACAP Receptor Inhibition Experiments

PC12 cells were adjusted to 1 × 10^3^ to 5 × 10^3^ cells/well on a 96-well plate and cultured for 24 hours and the PAC1-R inhibitor (PA-8: Professor Ichiro Takasaki, Faculty of Engineering, University of Toyama) [38]. VPAC1-R and VPAC2-R inhibitors (VIP6-28, V4508, Sigma-Aldrich) were added to the cell culture and incubated in a CO_2_ incubator (37°C, 5%) for 1 hour. PACAP38 10^−7^ M was added postincubation and cells were observed with or without the addition of inhibitors. A BZ-X710 optical microscope (Keyence) was used to count the total number of cells, and the number of cells with elongated protrusions (20 *μ*m or more) were counted.

### 2.6. Inhibitor Experiments

PC12 cells were adjusted to 1 × 10^3^ to 5 × 10^3^ cells/well on a 96-well plate and cultured for 24 hours, and 5 *μ*M CHIR99021 (252917-06-9, FUJIFILM Wako Pure Chemical Corporation), 5 *μ*M LY294002 (154447-36-6, FUJIFILM Wako Pure Chemical Corporation), 2.5 *μ*M H89, 2.5 *μ*M U0126, 2.5 *μ*M GF109203X, 10 *μ*M Y27632, and 5 *μ*M purvalanol A were added and placed in a CO_2_ incubator (37°C, 5%) for 1 hour, and PACAP 10^−7^ M was added postincubation. After culturing for 3 days, the culture was observed using an optical microscope BZ-X710 (Keyence) and the total number of cells and the number of cells producing protrusions (greater than 20 *μ*m and 20 *μ*m or less) were measured.

### 2.7. Extraction of Total Soluble Protein

For extracting the proteins, PC12 cells were washed twice with PBS followed by the addition of the LB-TT extraction solution (7 M (*w*/*v*) urea, 42 g; 2 M (*w*/*v*) thiourea, 15.2 g; 4% (*w*/*v*) CHAPS, 4.0 g; 18 mM (*w*/*v*) Tris-HCl (pH 8.0), 1.8 mL; 14 mM (*w*/*v*) Trizma base, 169.5 mg; 0.2% (*v*/*v*) Triton X-100; 0.2 mL 50 mM (*w*/*v*) DTT, 771.5 mg; 1% (*v*/*v*) pH 3-10 ampholyte, 1 ml; and two EDTA-free proteinase inhibitor (5892791001, Roche) tablets in a total volume of 100 ml) to lyse the cells. One (1) ml of LB-TT was quickly added to the culture dish (diameter 10 cm) and immediately mixed for 1 min at RT. Protein concentration was determined with a Pierce™ 660 nm Protein Assay Reagent (Thermo Fisher Scientific) using bovine serum albumin (BSA) as a standard and a DeNovix DS-11 spectrophotometer (DeNovix, Wilmington, DE, USA).

### 2.8. Two-Dimensional Gel Electrophoresis and Visualization of the Separated Proteins

Two-dimensional gel electrophoresis was performed according to the ATTO technical manual. Five (5) *μ*g of the protein sample was added to a precast agarose disc gel (pH 5–8, *φ*2.5 mm × 75 mm; ATTO, Tokyo, Japan), and one-dimensional electrophoresis was performed using a WSE-1500 dicRun-R (ATTO, Tokyo, Japan) at a constant voltage of 300 V for 210 min. After completion of the one-dimensional electrophoresis, agarose disc gels were fixed in a fixative solution (0.25% TCA) for 3 minutes. The gels were washed three times with distilled water for 1 min, replaced with new distilled water, and shaken gently for 2 h, at RT. The distilled water was discarded, and the cells were gently shaken in SDS equilibrium solution (50 mM Tris-HCl (pH 6.8), 1.6% SDS, 0.02% bromophenol blue, 8% Glycerol, and 20 mM DTT) for 10 min, followed by a two-dimensional electrophoresis step. The two-dimensional electrophoresis was performed using e-PAGEL(R) (E-D520L 5-20%) at a current of 20 mA/gel for 90 min, using a WSE-1150 PageRunAce (ATTO, Tokyo, Japan). Following 2-DE, proteins were transferred onto the PVDF membrane (Trans-Blot Turbo Midi PVDF, 0.2 *μ*M, Transfer Packs Kit; cat. no. 170-4157) using the Trans-Blot Turbo Transfer System (Bio-Rad) following the MIXED MW protocol, 25 V, 2.5 A, 7 min. Following the transfer of proteins on the PVDF membrane (as also confirmed by visualizing all the 10 colored molecular mass standards), it was incubated in 25 ml of blocking solution for 1 h under constant slow shaking at RT. Blocking solution was prepared by dissolving 5.0 g skim milk (Difco) in 100 ml 1X TTBS (10X TTBS: NaCl 80 g; 1 M Tris-HCl (pH 7.5) 200 ml; and Tween-20 10 ml/l). Blocking solution was decanted, and the membrane was washed once in 1X TTBS (5 min), followed by incubation in 10 ml of primary antibody solution (2 *μ*l rabbit anti-CRMP2 (cat. no. ab62661; Abcam), 3 *μ*l mouse anti-CRMP2 phospho T555 (cat. no. ab215742; Abcam), 3 *μ*l rabbit anti-CRMP2 phospho S522 (cat. no. ab193226; Abcam), and 3 *μ*l rabbit anti-CRMP2 phospho T514 (cat. no. ab85934; Abcam) for 1 h, as above). The membrane was then washed five times with 25 ml of 1X TTBS. After decanting the last TTBS wash, the membrane was incubated in 10 ml of secondary antibody solution (0.5 *μ*l of Amersham, ECL anti-rabbit IgG, HRP linked species-specific whole antibody (from Donkey); C=cat. no. NA 934; GE Healthcare) for 1 h, with slow shaking at RT. The 1X TTBS wash step was repeated five times. For image/band development, the luminol/enhancer and peroxide buffer solutions were mixed in a 1 : 1 ratio (Clarity Western ECL Substrate, cat. no. 170-5060, Bio-Rad) and spread over the membrane and incubated at RT for 5 min. Excess solution was drained by touching one end of the membrane on a Kimwipe paper towel, and the signal (crossreacting protein bands) was visualized on a ChemiDoc XRS+ imaging system (Bio-Rad).

### 2.9. Statistical Analysis

All experiments were repeated multiple times (six times unless stated otherwise). Data are presented as mean ± standard deviation. Microsoft Excel was used to analyze the data. Means were compared using one-way analysis of variance to confirm significance, and the Tukey method was used for multiple comparisons.

## 3. Results and Discussion

### 3.1. Expression of PACAP and Its Receptors in the PC12 Cells

The primary objective here was to confirm the presence or absence of receptor gene expression in PC12 cells; therefore, gene expression analysis of PACAP and its receptors in PC12 cells was examined. Semiquantitative RT-PCR, which is an established method with the right primer design and visualization of product/band on gel, was used, and after 45 PCR cycles, the PACAP, PAC1-R, VPAC1-R, and VPAC2-R mRNA were shown to be expressed. However, on reducing the PCR reaction time to 36 cycles, only a single band was confirmed for the PAC1-R. This suggested that PAC1-R is the main receptor involved in the neurite protrusion elongation action of PACAP38 in PC12 cells (Table 1). In the case of tissues, the difference in immunostaining can be clearly shown due to the difference in receptors, but in the case of cells, only similar images are obtained, so we confirmed the expression level by quantitative PCR in the present study.

### 3.2. Effect of PACAP38 Concentration on Neurite Protrusion Elongation

**Figure 1:**
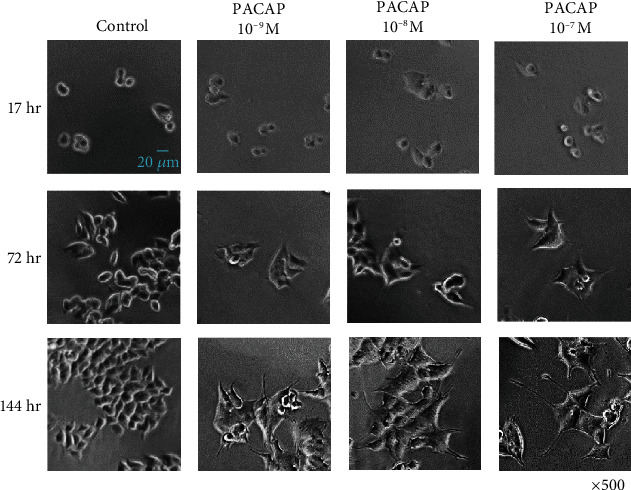
Changes in the PC12 cell neurite outgrowth with different concentrations of PACAP38. Experiments were repeated six times (*n* = 6).

**Figure 2:**
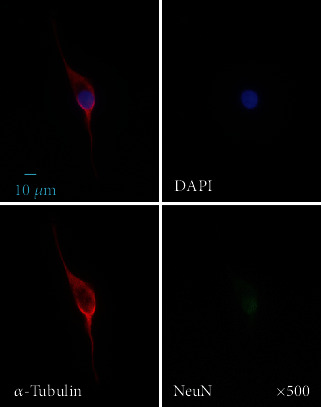
PC12 cell immunostaining. DAPI: blue; *α*-tubulin: red; NeuN: green. Experiments were repeated six times (*n* = 6).

**Figure 3:**
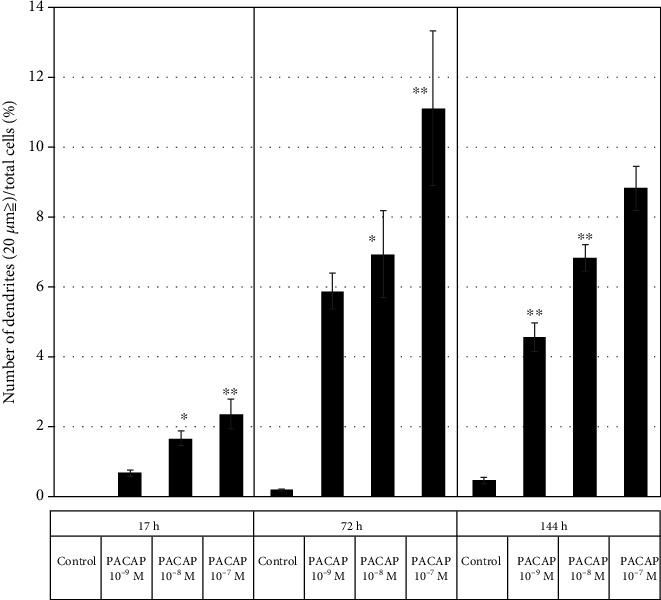
Change in the number of protrusions under different PACAP concentrations in the PC12 cells. Control: PACAP not added (*n* = 6); PACAP: PACAP38 concentrations added as indicated (*n* = 6). ^∗^*p* < 0.05 vs Control; ^∗∗^*p* < 0.01 vs Control (Tukey test). Experiments were repeated six times (*n* = 6). Data are presented as the mean ± SD.

In order to investigate the optimum concentration of PACAP38 under which the PC12 cells show elongate protrusions, various concentrations of PACAP38 were added to PC12 cells and changes in the cells were observed (Figure 1). Immunostaining was used to observe the protrusions in the PC12 cells and their elongation by PACAP38. In the PACAP-added PC12 cells, NeuN specifically localized in the nerve cell nucleus, whereas *α*-tubulin expression was confirmed in the cell body and processes. This suggests that the protrusions elongated by PACAP38 are indeed the neurite protrusions (Figure 2). By measuring the protrusion elongation cell ratio of PC12 cells at various concentrations of PACAP38 over time, we could confirm the role of PACAP38 in causing protrusion elongation; the number of cells in the PACAP 10^−7^ M addition group confirmed that the protrusion elongation was significantly increased as compared to that in the control group (Figure 3). Results are the mean of different well data, and error bars are shown in SD (Figure 3).

### 3.3. Changes in Neurite Protrusion Elongation Action by PACAP Receptor Inhibitors

**Figure 4:**
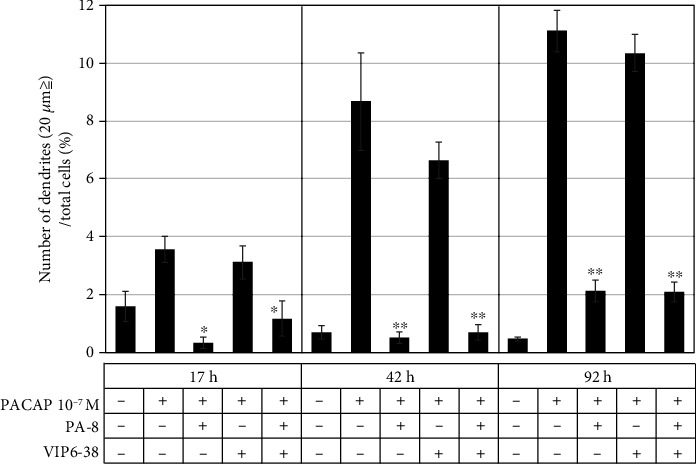
Effect of the PACAP receptor inhibitor on neurite outgrowth in PC12 cells. ^∗^*p* < 0.05 vs PACAP 10^−7^ M; ^∗∗^*p* < 0.01 vs PACAP 10^−7^ M (Tukey test). Experiments were repeated six times (*n* = 6). Data are presented as the mean ± SD.

In order to identify the receptors involved in the action of PACAP38 in the PC12 cells, PA-8, which is a specific PAC1-R inhibitor, and VIP6-28, which is a specific VPAC2-R inhibitor, were added and changes in the protrusion elongation action by PACAP38 was observed. The protrusion elongation effect of PACAP38 was significantly suppressed in the presence of PA-8, but not with VIP6-28. This data suggested that the protrusion elongation action of PACAP38 is mediated through the PAC1-R (Figure 4).

### 3.4. Observation of Neurite Protrusion Elongation Using Inhibitors and Activators

#### 3.4.1. Examination of the GSK-3*β* Pathway

**Figure 5:**
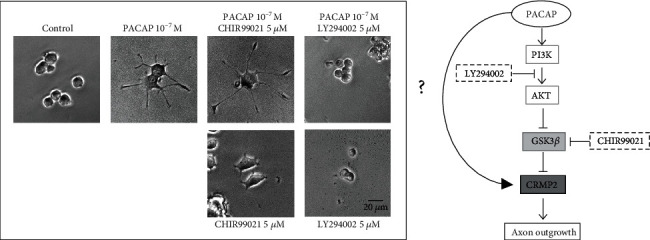
Changes in neurite outgrowth in the PC12 cells due to GSK-3*β* inhibition and activity. Control: PACAP free; CHIR99021: GSK-3*β* inhibitor; LY294002: GSK-3*β* activator. Treatment with PACAP38 was done for 72 h.

To clarify whether CRMP2 acts directly on PACAP38 or through GSK-3*β* as part of the mechanism of PACAP38-induced neurite outgrowth, we observed neurite outgrowth in the presence of GSK-3*β* inhibitors and activators. The addition of CHIR99021, an inhibitor of GSK-3*β*, promoted neurite outgrowth induced by PACAP38, while the addition of LY294002, an inhibitor of PI3K (phosphoinositide 3-kinase) upstream of GSK-3*β* and activator of GSK-*β*, inhibited neurite outgrowth induced by PACAP38. In this experiment, a neurite outgrowth of less than 20 *μ*m was observed under the conditions where only CHIR99021 or LY294002 was added but most of the neurite outgrowth was greater than 20 *μ*m when only CHIR99021 was added. However, when LY294002 was added, there was almost no elongation of more than 20 *μ*m. These results suggest that the protrusion elongation effect of PACAP38 is mediated by GSK-3*β* (Figure 5).

Although PACAP38-induced PC12 neurite outgrowth was thought to be mediated by the GSK-3*β* pathway, previous studies have shown that the signaling cascade from the PACAP receptor PAC1 moves mainly via the AKT pathway, the cAMP/PKA pathway, the cAMP/ERK pathway, and also the PLC/PKC pathway [39]. CRMP2 is known to be regulated by the PI3 kinase/Akt/GSK-3*β* signaling pathway [31]. GSK-3*β*, in addition to the AKT pathway, is also involved in the cAMP/PKA, cAMP/ERK, and PLC/PKC pathways [31, 39]. GSK-3*β* has been reported to be regulated by the cAMP/PKA, cAMP/ERK, and PLC/PKC pathways in addition to the AKT pathway [40, 41].

**Figure 6:**
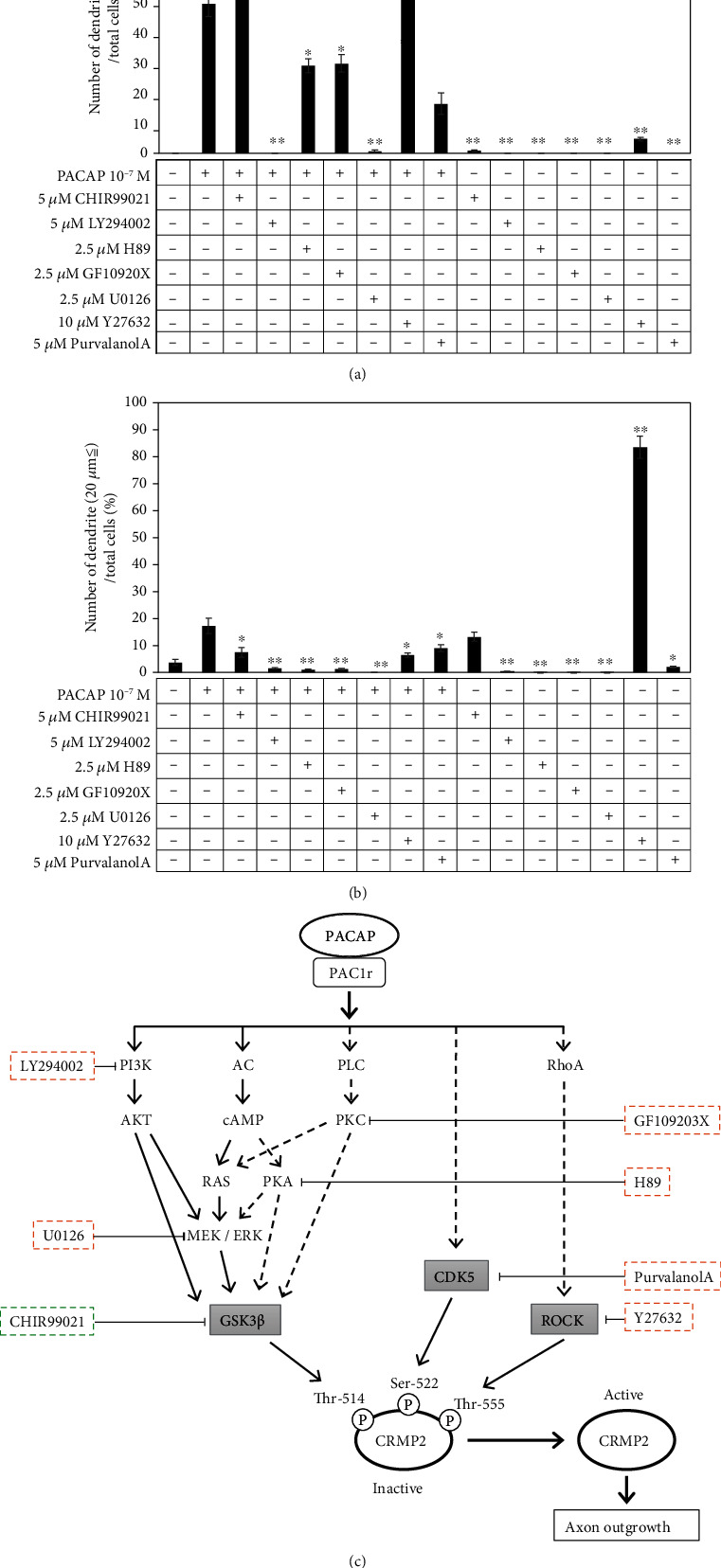
(a) Examination of pathways involved in protrusion extension (20 *μ*m or more) by PACAP in inhibitor experiments. ^∗∗^*p* < 0.01 vs PACAP 10^−7^ M. Control: PACAP free; CHIR99021: GSK-3*β* inhibitor; LY294002: GSK-3*β* activator; H89: PKA inhibitor; U0126: MEK/ERK inhibitor; GF109203X: PKC inhibitor; purvalanol A: CDK5 inhibitor; Y27632: rock inhibitor. (b) Examination of pathways involved in protrusion extension (less than 20 *μ*m) by PACAP in inhibitor experiments. ^∗∗^*p* < 0.01 vs PACAP 10^−7^ M. Control: PACAP free; CHIR99021: GSK-3*β* inhibitor; LY294002: GSK-3*β* activator; H89: PKA inhibitor; U0126: MEK/ERK inhibitor; GF109203X: PKC inhibitor; purvalanol A: CDK5 inhibitor and Y27632: rock inhibitor. (c) Examination of the pathways involved in protrusion extension in PC12 cells by PACAP using inhibitor experiments. →: pathways involved in process extension by PACAP; ⤏: pathways that are less involved in PACAP-induced protrusion extension.

From the results of experiments using inhibitors (Figures 6(a) and 6(b)), it was confirmed that the addition of the AKT inhibitor LY294002 and the cAMP/ERK inhibitor U0126 almost completely suppressed the effect of PACAP38 on protrusion elongation. Therefore, under the present experimental conditions, the AKT pathway and the cAMP/ERK pathway were confirmed to be the major processes responsible for the neurite outgrowth effect of PACAP38 in PC12 cells.

#### 3.4.2. Examination of the CDK5 and Rho/ROCK Pathways

In addition to GSK-3*β*, CDK5 and RhoA have been reported to phosphorylate CRMP2. Sema3A activates Cdk5 and GSK-3*β*, and Cdk5 phosphorylates CRMP-2 with serine 522 [35, 42]. With regard to the RhoA, activation of RhoA disrupts neurite outgrowth in primary neurons [36, 43] and myelin-related glycoproteins inhibit axon regeneration by a Rho-kinase-dependent mechanism [37, 44]. As a result of conducting experiments with the CDK5 and Rho/Rock pathway inhibitors and confirming the presence or absence of protrusion elongation by PACAP38, it was confirmed that the protrusion elongation action of PACAP was promoted when the Rho/Roc inhibitor Y27632 was added. When only Y27632 was added, many PC12 cells with protrusions of 20 *μ*m or less were observed but protrusion elongation of 20 *μ*m or more was almost negligible. Therefore, it can be suggested that Rho/Rock suppression by Y27632 alone does not cause a protrusion elongation effect. On the other hand, the addition of purvalanol A, a CDK5 inhibitor, slightly suppressed the protrusive elongation effect of PACAP38. From these results, the protrusion elongation action by PACAP38 is involved in both the CDK5 and Rho/Rock pathways but it can be suggested that rather than CDK5 phosphorylating CRMP2, PACAP promotes protrusion elongation via the CDK5 (Figures 6(a) and 6(b)).

From the results of the above inhibitor experiments, it can also be suggested that the main pathways for neurite protrusion elongation by PACAP are the AKT pathway and the cAMP/ERK pathway and that Rho/Roc inhibition was considered to further promote the neurite protrusion elongation action by PACAP38. However, for CDK5, if CRMP2 phosphorylation by CDK5 acts for the inactivation of CRMP2, the addition of purvalanol A should result in promoting the neurite protrusion elongation action by PACAP38 addition, but in this experiment, the neurite protrusion elongation action was suppressed. Elimination of Cdk5-mediated CRMP2 Ser-522 phosphorylation reduces the density of dendrite spines in hippocampal neurons in the mouse hippocampus [45] and Cdk5-inhibition of CRMP2 phosphorylation in Ser522 in the optic nerve. There are reports that it leads to stabilization and regeneration of axons after nerve damage [46]. In addition, class 3 semaphorins have been reported to mediate dendrite growth in adult neonatal neurons through the Cdk5/FAK pathway [47]. It is implied that the CDK5 activation may be required for the neurite protrusion elongation action by PACAP38 in the PC12 cells (Figure 6(c)).

### 3.5. Relationship between CRMP2 Phosphorylation and Neurite Protrusion Elongation

**Figure 7:**
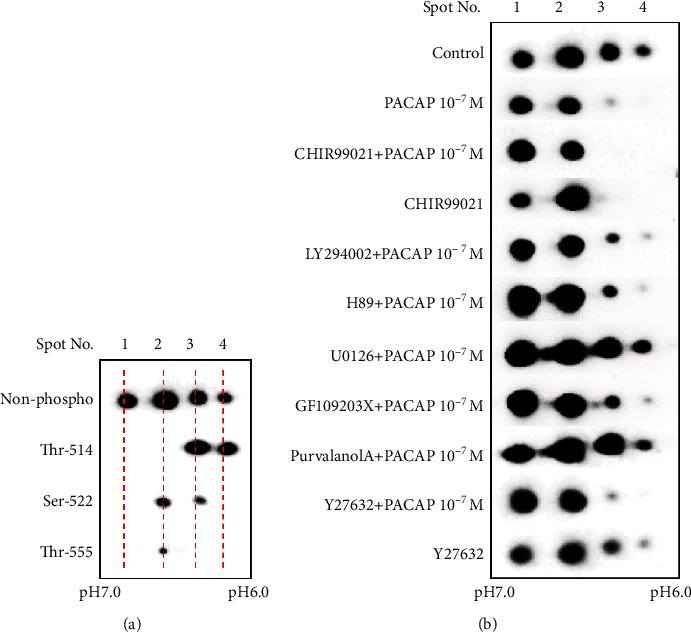
Examination of pathways involved in the CRMP2 phosphorylation and dephosphorylation by PACAP in the inhibitor experiments. (a) Antibody used: non-phosphor: anti-CRMP2 (ab129082); Thr-514: anti-CRMP2 phospho Thr-514 (ab85934); Ser-522: anti-CRMP2 phospho Ser-522 (CK6200); Thr-555: anti-CRMP2 phospho Thr-555 (CK6200). (b) Antibody used: anti-CRMP2 (ab129082); control: PACAP free; CHIR99021: GSK-3*β* inhibitor; LY294002: GSK-3*β* activator; H89: PKA inhibitor; U0126: MEK/ERK inhibitor; GF109203X: PKC inhibitor; purvalanol A: CDK5 inhibitor; Y27632: Rock inhibitor. Two-dimensional gel electrophoresis was performed as described in Cell Model and Methods.

Although CRMP2 phosphorylation and dephosphorylation are important for neurite protrusion elongation, the relationship between the phosphorylation/dephosphorylation action of CRMP2 in PACAP38 and neurite protrusion elongation is not clear. In particular, CRMP2 has many phosphorylation sites, and therefore, a two-dimensional gel electrophoresis analysis experiment was conducted to clarify which phosphorylation site might be involved and, thus, is important for the neurite protrusion elongation by PACAP38 (Figure 7).

As a result of the two-dimensional gel electrophoresis analysis (Figures 7(a) and 7(b)), four (4) spots were detected with the CRMP2 antibody. When Thr-514-, Ser-522-, and Thr-555-phosphorylated CRMP2 antibodies were used to confirm crossreacting phosphorylation spots, spot 2 was shown to be Ser-522 and Thr-555 and spot 3 was shown to be Thr-514 and Ser-522. It was confirmed that spot 4 was Thr-514. Spots 2, 3, and 4 abundance reduced with PACAP38 addition compared to the control. With the addition of PACAP38 + CHIR99021, which promoted increased neurite protrusion elongation, spot 2 decreased and spots 3 and 4 disappeared. Spot 4 disappeared in PACAP38 + Y27632, which also showed more promotion of the neurite protrusion elongation. Furthermore, the addition of PACAP38 + LY294002 and PACAP38 + U0126, which eliminated the protrusion elongation action, did not cause a reduction in spots 3 and 4. Furthermore, spot 4 decreased in PACAP38 + H89, PACAP38 + GF109203X, and PACAP38 + Purvalanol A in which protrusion elongation was slightly suppressed. From the above results, it was considered that the reduction of spots 3 and 4 is important for promoting neurite protrusion elongation by PACAP38, i.e., the Thy-514 dephosphorylation of CRMP2 is essential. However, although spots 3 and 4 disappeared as in the case upon addition of CHIR99021, neurite protrusion elongation of 20 *μ*m or more could not be confirmed. We cannot explain this observation at present, and a more detailed analysis will be required to understand it better. However, the ratio of spot 1 to spots 2, 3, and 4 was also considered to be important for neurite protrusion elongation of 20 *μ*m or more. It was considered that the lower the ratio of phosphorylated CRMP2, the more that the protrusion elongation of 20 *μ*m or more was promoted.

The neuropeptide PACAP is known to be involved in the neurite protrusion elongation action, but the mechanism of action has not been clarified. Therefore, PC12 cells, which are nerve-like cultured immortalized cells, were used to elucidate the mechanism of action. It was hypothesized that CRMP2 is involved in the protrusion extension action by PACAP38 but it was unclear what pathway was critical. From the results of this inhibition experiment, it was confirmed that the AKT pathway and the cAMP/ERK pathway mediated by the PAC1 receptor are the main pathways in the process elongation action by PACAP38. Furthermore, PACAP38 was found to promote neurite protrusion elongation by promoting dephosphorylation of CRMP2. The main pathways for dephosphorylation of CRMP2 have been reported to be via GSK-3*β*, Rho/Rock, and CDK5. However, in this study, results showed that the neurite protrusion elongation effect by PACAP38 was further promoted by dephosphorylation of CRMP2 by suppressing GSK-3*β* and Rho/Rock but the neurite protrusion elongation effect by PACAP38 was suppressed by CDK5 inhibition. CDK5 is said to be involved in the phosphorylation of Ser-522 in CRMP2, but inhibition of CDK5 does not reduce the spots of Ser-522 by two-dimensional electrophoresis, and spot 3 phosphorylation and spot 4 phosphorylation of Thy-514 were increased. CDK5 inhibition results in Ser9 dephosphorylation and in vivo activation of GSK-3*β* [48, 49]. Cdk5 activity is also associated with axonal and neurite growth [50]. In addition, Cdk5^−/−^ mice have been reported to exhibit defective axonal elongation [51, 52]. From the results of this experiment, GSK-3*β* activation by CDK5 inhibition and increased phosphorylation of Thy-514 of CRMP2 indicate that CDK5 activity may also be important for the neurite protrusion elongation action by PACAP38.

Aware of the limitations and the need for further research, we are currently investigating the molecular mechanism of CRMP2-mediated neurite protrusion elongation by PACAP38 using DNA microarray and shotgun (LC-MS/MS) proteomic analysis and will further examine whether phosphorylation of CRMP2 by PACAP38 is effective for axon regeneration after injury. In recent years, the inhibition of CRMP2 phosphorylation has been shown to be effective in various diseases such as Alzheimer's disease [53], spinal cord injury [54], amyotrophic lateral sclerosis [55], and optic nerve injury [46]. Studies of PACAP, which inhibits CRMP2 phosphorylation, might pave the way towards the development of new drugs and therapies.

## Figures and Tables

**Table 1:** Primer combinations used for semi-quantitative RT-PCR (*Rattus norvegicus*).

Accession (gene)	Nucleotide sequence (5′-3′)	Nucleotide sequence (5′-3′)	Product size (bp)	Gene name
NM_016989	CTGTTGGTCTACGGGATAAT	CTACAAGTACGCTATTCGGC	498	*PACAP (Adcyap1)*
NM_001270579	TTGCAAGATGTCAGAACTATCCA	GAAGTAACGGTTCACCTTCCAG	259	*RAC1-R (Adcyap1r1)*
NM_012685	AAATGGTCTTCGAACTTGTCGT	GGAGTGTGTCCCTATGAAAAGC	373	*VPAC1 (Vipr1)*
NM_017238	CACTAGTGATGGGTGGTCGG	GCCAGTAGAAGTTCGCCATG	399	*VPAC2 (Virp2)*
NM_017008	CCTGTGACTTCAACAGCAACTC	GGCCTCTCTCTTGCTCTCAGTA	213	*GAPDH*
NM_031144	TGACGGTCAGGTCATCACTATC	GGCAGTAATCTCCTTCTGCATC	229	*Actb*

## Data Availability

All data are publicly available; and data or methods or primers required will be shared fully.

## Abbreviations

CRMP2: Collapsing response mediator protein 2
GSK-3*β*: Glycogen synthase kinase-3*β*
PACAP: Pituitary adenylate cyclase-activating polypeptide
PC12: Rat adrenal-derived pheochromocytoma cell line
PMCAO: Permanent middle cerebral artery occlusion
VIP: Vasoactive intestinal polypeptide.
